# Disaster preparedness of Australian hospital networks: a qualitative study with key actors

**DOI:** 10.1136/bmjopen-2025-106500

**Published:** 2026-02-22

**Authors:** Faran Shoaib Naru, Kate Churruca, Janet C Long, Mitchell N Sarkies, Jeffrey Braithwaite

**Affiliations:** 1 Australian Institute of Health Innovation (AIHI), Macquarie University, Sydney, New South Wales, Australia; 2 School of Health Sciences, The University of Sydney, Sydney, New South Wales, Australia

**Keywords:** Risk management, Capacity Building, Public Hospitals

## Abstract

**Abstract:**

**Objective:**

Disasters can have a disproportionate impact on highly vulnerable hospitalised patients. Managers preparing hospital networks for disasters play an important role in enhancing networks’ readiness by creating disaster plans and imparting that knowledge through training and simulation exercises. The objective of this research was to uncover how those working in disaster preparedness roles in Australian hospital networks perceived the challenges that they face while ensuring adequate preparation for disasters.

**Design:**

A qualitative study design was employed which involved purposive sampling of Australian hospital network professionals responsible for disaster preparedness. Thematic analysis of data collected through individual online interviews generated prominent challenges of disaster preparedness in Australian hospital networks.

**Setting:**

Local hospital networks across Australia

**Participants:**

Twenty-six disaster preparedness managers, including hospital executives, disaster managers, emergency management coordinators and business continuity managers from 23 hospital networks located in five Australian states and one territory, participated in semi-structured online interviews. Interview transcripts were coded through an iterative inductive thematic analysis process to synthesise the predominant challenges faced by these participants when preparing their hospital networks for disasters.

**Results:**

Participants reported four challenges: staff’s limited interest in preparedness, budgetary constraints, staffing issues and ambiguous relationships with state and national health departments. They also presented four related solutions: capitalising on interest after disasters, attracting funding with evidence from prior disasters, facilitating staff’s availability for disaster training and specifying network-government relationships for accountability.

**Conclusion:**

Disasters, although infrequent, are known to occur and can be catastrophic, yet those working in hospital network disaster preparedness roles encounter limited availability of wider staff for training and low interest in disaster planning. The sudden onset of a disaster can take a heavy toll on patients if hospitals’ staff are not sufficiently trained in disaster response or are not aware of the disaster plan. By identifying the perceptions of managers to disaster preparedness, this research presents specific challenges that hospital networks can address to improve awareness and preparation.

**STRENGTHS AND LIMITATIONS OF THIS STUDY**

- Australian hospitals spread across a vast area of 7.6 million square kilometres face distinct disaster risks. Participants represented six out of eight Australian states and territories, and these six states and territories contain 98.4% of Australia’s population. This first Australia-wide study synthesises all hospitals’ challenges to enable an all-hazards approach to preparedness.
- Purposive sampling of disaster preparedness experts involving a lengthy and comprehensive participant recruitment process that reached out to 128 local hospital networks of all eight Australian states and territories allowed collection of largely inaccessible data.
- Inductive thematic analysis allowed collection of common challenges and potential solutions articulated by experts preparing diverse hospitals for disasters.
- Semi-structured interviews’ limitation of social desirability bias was mitigated through additional reframed questions probing participants on the research problem.

## Background

 Australia has a high-performing health system, with good access to healthcare services and a population that generally experiences positive health outcomes.^1^ Australia is also a disaster-prone country where disasters have impacted hospitalised patients. When hospitals in Cairns, Australia were threatened by a hurricane in 2011, hospitalised patients were evacuated without sufficient back up of medicines or oxygen, endangering the lives of those at risk of a deterioration in condition.^2 3^ When a hospital in Ballina, Australia was threatened by a flood in 2022, patients receiving care for terminal illness or cognitive decline were relocated to a school with limited healthcare equipment.^4^ Examples of disasters from just the last 6 years include bushfires that forced closures of two hospitals in 2019,^5^ a COVID-19 outbreak that closed a hospital in 2020,^6^ floods that led to a hospital evacuation in 2022^7^ and a bushfire forcing another hospital evacuation in 2023.^8^ Almost every year, Australian hospital patients have been impacted by disasters, many of which are directly or indirectly attributable to climate change. Australia’s Royal Commission into National Natural Disaster Arrangements in 2020 predicted that climate change will increase the intensity and frequency of disasters,^9^ which in turn may have serious consequences for hospital patients in Australia. Besides extreme weather events threatening hospital infrastructure, mass casualty incidents can also result in resource shortfalls among other challenges. To ensure patient safety when the number of catastrophic events is expected to increase, hospital networks need to both reduce the risk of disaster damage and proactively implement preparedness measures. United Nations Office for Disaster Risk Reduction defines disaster preparedness as “*the knowledge and capacities developed to effectively anticipate, respond to and recover from the impacts of likely, imminent or current disasters*”.^10^

Although Australian federal and state governments can provide support if needed, hospital disaster preparedness, response and recovery falls within the purview of local hospital networks which are legal entities that manage groups of public healthcare facilities.^11 12^ In Australia’s most populous state, New South Wales (NSW), the state health disaster plan designates the state health department as the primary agency responsible for disaster response, but rests the entire responsibility for local emergency management with hospital networks.^13^ This policy directive of the NSW state government tasks Disaster Managers of those hospital networks with development and maintenance of preparation strategies and adds that disasters must initially be managed within hospital network’s resources.^13^ Section 313 of NSW HEALTHPLAN states that hospital networks must undertake risk assessment to develop control plans, response and surge plans, and business continuity and recovery plans for bushfires, storms, floods, hospital evacuations and utility disruptions.^13^ The frequency and intensity of these and other disasters in Australia are such that any under-preparedness could have direct negative consequences for hospitalised patients. Stipulation of preventive planning may not achieve optimum risk reduction outcomes if there are unidentified challenges to the implementation of preparedness measures.

Many healthcare organisations and services have plans and practise or simulate readiness exercises or coping mechanisms to reduce disaster risks. A recent survey of Australian hospital networks’ disaster preparedness levels reported that 98% of participating hospital networks were conducting readiness exercises for evacuation preparedness and most hospital networks had optimal logistical backups of water, fuel, generators, medical gases and sterilised equipment.^14^ However, many participating hospital networks also revealed limited preparations for a range of critical issues, prominent among which was patient tracking in hospital evacuations.^14^ Limited implementation of patient tracking measures can result in substantial harm for elderly patients with complex medication schedules or mobility and cognitive deficits.^15^ Elderly patients in Australia are also vulnerable as they formed the bulk of a rural hospital evacuation conducted in 2022,^4^ yet implementation of patient tracking measures was found to be limited in Australian hospitals.^14^ The limited implementation of patient tracking measures is a known challenge when undertaking hospital evacuations, such as after the 2012 Hurricane Sandy when patients in the US were evacuated without accompanying medical records so they could not be traced by their families,^16^ and in the Netherlands when over a hundred patients could not be tracked for up to 4 days after an aeroplane crash.^17 18^ Despite multiple systematic reviews of hospital disaster risk reduction and WHO checklists’ recommendation of patient tracking initiatives among other implementable measures, the measure remains underutilised, requiring a thorough investigation of the challenges that may be restricting implementation of this and other key preparedness measures.^19^,^25^

Certain hospital preparedness measures have been identified as important in past disaster events and in academic literature. However, these measures are not consistently applied in hospital preparedness internationally. Hospital patients are vulnerable and face disproportionate risks in the face of human-induced disasters or disasters caused by vulnerability to natural hazards. Mitigating their post-disaster mortality and morbidity risks involves ensuring that a hospital is not compromised by a disaster, is able to maintain the delivery of safe care despite disaster damage, or safely evacuates patients if the hospital becomes incapacitated. Planning of such post-disaster functions involves timely implementation of potentially lifesaving preparedness measures. However, inadequate implementation of measures can limit hospital network’s preparedness, leaving hospitals and their patients vulnerable to disaster damage, mortality and morbidity. Considering the importance of preparing Australian hospitals for disasters, research was needed to understand both the difficulties that hospital networks’ disaster managers face and the solutions that they innovate. It is important to explore what might be restricting implementation of widely reported preparedness measures in Australian hospital networks and the local solutions to those issues. This research aimed to uncover the challenges faced by hospital network staff that are responsible for implementation of measures aimed at reducing the risk of disaster impacts on hospitals and their patients, and overall disaster preparedness.

## Methods

### Study design

A qualitative research design was used which involved semi-structured interviews with personnel that prepare hospital networks for disasters. This study is reported according to the consolidated criteria for reporting qualitative research checklist^26^ (attached as online supplemental appendix 1). The research was developed and conducted in accordance with Australia’s National Statement on Ethical Conduct in Human Research.^27^ The ethical approval for this research was granted by the Medicine and Health Sciences Subcommittee of Macquarie University’s Human Research Ethics Committee: Reference No: 520221213642123, Project ID: 12136. Although patients and the public were not directly involved in this research, our research institution frequently incorporates the feedback of patients and catchment populations in almost all our health services research, and that feedback also informed the conceptualisation of this research.

### Participant recruitment

Australian states and territories have established 132 hospital networks that manage public hospitals across all six states and two territories of Australia, spread over diverse topographies ranging from arid regions disposed to bushfires, to tropical regions prone to cyclones.^11^ Out of these 132 networks, four did not provide round-the-clock emergency services. Excluding these four, all remaining 128 hospital networks that manage 293 Australian public hospitals providing 24/7 emergency care were invited to participate in this research. This involved targeting personnel who had clear mandates of disaster management, emergency preparedness or business continuity within their hospital networks. All these networks were telephoned to recruit staff members that either had relevant designations or looked after these domains as senior executives of the hospital network. Participant information and consent forms, explaining the objective and methodology of the research, were included in both the initial research invitation emails to the 128 hospital networks and the reminder emails.

### Data collection

An interview schedule was developed to elicit open discussions on hospital networks’ disaster preparedness challenges. The research team adapted the interview schedule from a piloted questionnaire that had been used to study disaster preparedness in Australian hospital networks,^14^ and so the developed interview schedule was not piloted with potential participants. The interview questions were not shared with participants beforehand, and they elicited discussions on various disaster preparedness functions to collect data on implementation of disaster preparedness measures. The discussed functions included evacuation preparedness and patient tracking, staff credentialing, point of care testing, electronic medical record connectivity and implementation of disaster triage.^1920 28^,^30^ The interviews collected data on overarching challenges and solutions to the implementation of these and other hospital network disaster preparedness functions. The first author (PhD), who is a male higher degree health services researcher with 15 years’ experience in disaster management, conducted all interviews between 14th March and 24th April 2024. All interviews were conducted, recorded and transcribed on Microsoft Teams and no repeat interviews were carried out. The interviews were intended to take approximately 30 min, and almost all participants used the entire time.

### Data analysis

Interviews transcribed by Microsoft Teams’ auto-transcription service were checked for accuracy against recordings. Entire transcripts were also returned to participants for comments and corrections. The participants were not invited to provide feedback on the findings. After removing transcription errors, identifiable information in the transcripts was replaced with re-identifiable codes. Details on re-identifiable codes were separated to protect the privacy and confidentiality of participants. A preliminary coding framework was inductively developed in the process of conducting interviews and then while checking transcripts to capture recurrent themes in the data (eg, staff shortages, budgetary constraints). The de-identified transcripts were then imported into NVivo V.14 for thematic analysis. The transcripts were read in their entirety and coded line by line. Similar codes were merged into overarching themes, for example, all codes on financial limitations such as limited funds for training, or limited financing for emergency preparedness section within the hospital network, were merged into the overarching theme of budgetary concerns. The earlier coding framework was expanded and refined during the detailed review of transcripts (eg, by adding a theme for unclear coordination with state and national governments). The thematic analysis was conducted by the first author, but KC, JCL and JB continuously evaluated and classified the findings as the analysis proceeded. All authors formally ratified the final themes emerging from the analysis.

## Results

### Participants

**Figure 1:**
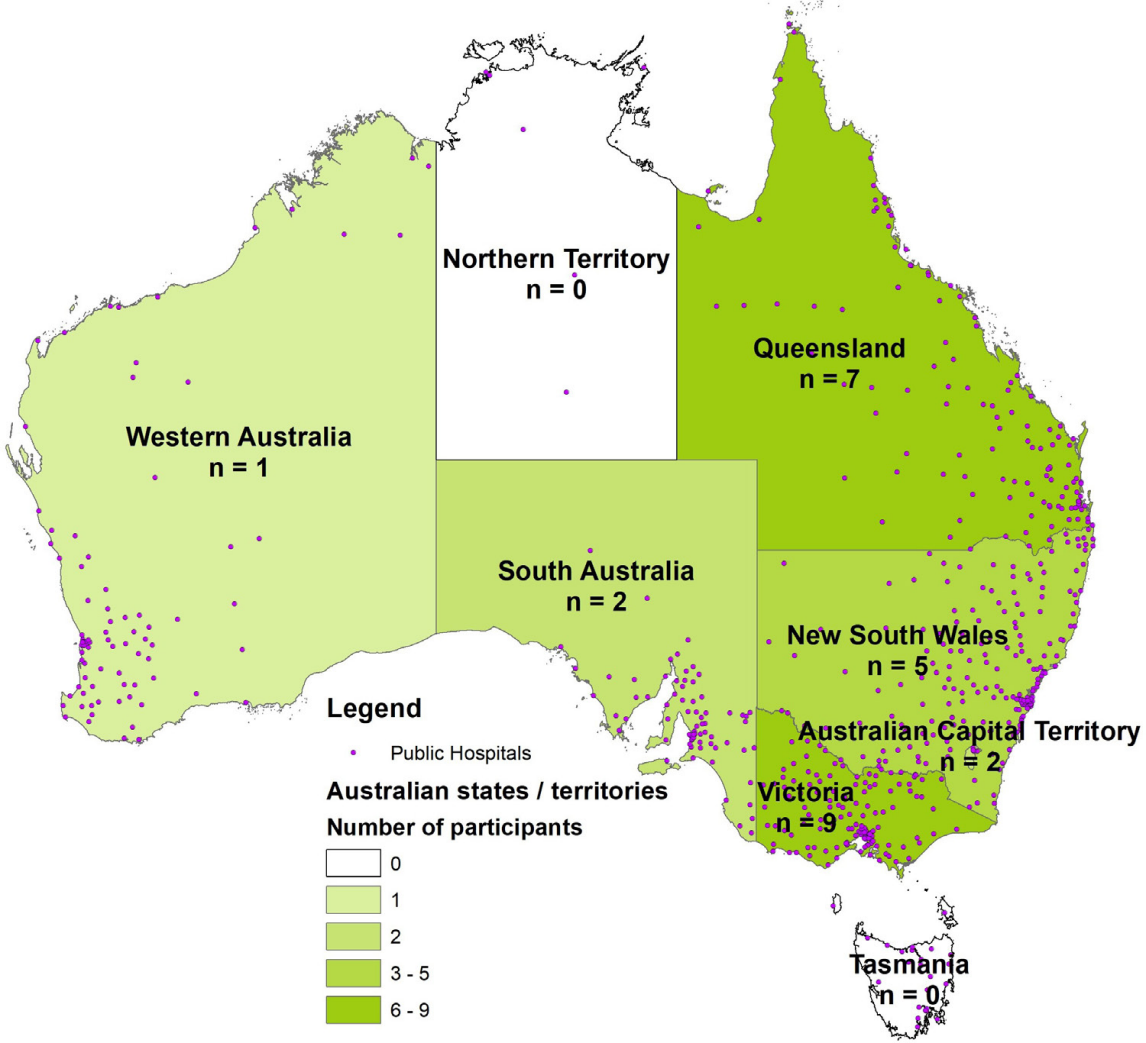
State-wise breakup of research participants and locations of Australian public hospitals. Data source: Public Hospitals Feature Layer by Department of Health and Aged Care.^54^

A total of 26 experts from 23/128 (18%) hospital networks providing emergency care, situated in five Australian states and one territory, were interviewed. Three hospital networks were represented by two disaster preparedness specialists, and the remaining 20 were one-on-one interviews. Data saturation was achieved through these multiple interviews. Participants of different states prepared their hospital networks for different hazards, for example, hospitals in northern Queensland are located in high cyclone risk areas, those in northern NSW are in high flood risk areas. The risk of bushfire damage, however, is faced by hospitals across Australia. Most hospital networks participating in this research had been affected by bushfires in 2019–2020 and then COVID-19 in succession. A number of participating hospital networks had also been affected by 2022 or earlier floods; therefore, almost all participants’ responses were informed by their practical experiences in disaster response. The number of participants from different states was roughly proportional to the number of public hospitals located in the state. The following map (figure 1) presents the state-wise breakup of research participants and shows how states with higher number of public hospitals also had a higher number of hospital network disaster managers participating in this research:

The most common designations of participants were Disaster Manager, Emergency Preparedness Manager or Director of Emergency Management. Table 1 provides a breakdown of participants’ main roles in their local hospital networks:

**Table 1:** Participants’ roles within their local hospital networks

Designations of participants	Number of participants
Roles dedicated to disaster management (eg, Disaster Manager, Emergency Preparedness Manager, Director of Business Continuity)	20
Executive roles in hospital management (eg, Executive Director, Quality and Safety, Chief Operating Officer)	3
Broader corporate roles with additional responsibility of disaster management (eg, Health and Safety Manager, Director of Corporate Services)	3
**Total participants**	**26**

### Challenges to the implementation of hospital disaster preparedness measures

The inductive thematic analysis led to the identification of the following four overarching themes: limited interest in preparedness, budgetary constraints, staff shortages and ambiguous relationship with state and national health departments.

#### Hospital network staff’s limited interest in disaster preparedness

Several participants shared that a major challenge for them was to get hospital network staff, particularly their executives, interested in disaster preparedness. A participant explained the lack of interest in following words: “*one of the challenges for myself is to create relevance of these plans and an interest in developing them and practising them*” (Participant 10). Another participant indicated that it is difficult to advocate disaster preparedness as an important aspect because you cannot put dollar values on its benefits:

People are very reluctant or don’t understand the importance of Emergency Management … it’s our job to help them appreciate the benefits, but that can be very difficult when it’s hard to quantify. So, if you have an incident occur, through things like insurance claims … it’s very easy to quantify how much that particular incident cost. But it’s very difficult, prior to then, to say if you spend so much money on preparing for that, you will save this much money on it actually occurring (Participant 9).

Participants reported that it is frustrating for them to see the hospital network staff or leadership being indifferent towards preparation or assuming that someone else will take care of the disaster. A participant mentioned the indifferent attitude in the following words: “*a ‘(it) will be all right’ attitude that somebody will kind of help me get through, or we’ll get through, I feel like what we see in the community is reflected within our staff*” (Participant 20). Another participant shared that owing to network executives’ busy schedules, the participant often observed only nominal compliance from leadership, which can have negative consequences for hospitals.

As there’s something in place, they generally consider that we’re compliant, and so the quality of that, to get to the gold standard or even the silver standard isn’t relevant for the purpose of that health service directive to our executive, as long as we’ve got something in place, then we meet it … So, there are the requirement to have a disaster management plan, right? And my executive goes, yeah, we’ve got a disaster management plan. I said yeah, but it’s years old and it’s completely out of date, so it’s not really even useful. It doesn’t matter. We’ve got one, tick it off … that all falls back to the lack of capacity and the lack of support (Participant 16).

Another participant highlighted hospital network leadership’s limited interest in disaster preparedness training and explained that it does not take precedence over other mandatory training.

One of the other challenges to note with training in a hospital setting, is that there is an enormous amount of mandatory training that everybody, including an admin staff, need to do and it’s competing with that mandatory training time and trying to say well actually Disaster management should be mandatory, but it can’t be because it’s not considered that by our executives (Participant 16).

Participants shared how they have been attempting to get the hospital network executives interested in disaster preparedness. A participant acknowledged the difficulty of eliciting hospital network executives’ interest in comprehensive disaster plans:

Incident command system procedures are 170 pages, so the first 40 pages are instructions about how to use the manual and the following 130 are action cards and templates … but it can be a little bit overwhelming when the executives get a copy of that and they’re like 170 pages, I don’t have time to read 170 pages. So, I think trying to synthesise that information and make it more readable and understandable to the executive so that they can understand what their role would be, will be one of the challenges (Participant 1).

A participant argued that their job entails making considerable efforts in convincing executives on the importance of disaster preparedness: “*There is some good practice guides out there, but it really does depend on your executive finding this to be really important and then prosecuting the argument because at the end of the day, we are subject to our executives’ whim, I guess”.* (Participant 17). Another participant further pointed out that the lengthy nature of disaster plans was a problem, making it difficult to attract and sustain staff interest in the context of the myriad of other important activities of their role:

We now have procedures that are 50 pages long … if I’m a clinician on the floor, I’m looking at my patient, I’m looking after my colleagues, I’m looking after my department and I’m looking after my division. Disaster and Emergency Management is probably the 100th thing that I’m thinking about in a day … It’s not at the forefront, so for me, a real challenge is relevance. How do I make it relevant for them? How do I get them to understand that it’s all about protecting them in the first place and protecting their patients (Participant 20).

#### Budgetary constraints

Several participants mentioned lack of funds as an impediment to preparing for disasters, stockpiling resources and training hospital network staff for potential disasters. A participant said: “*The health services are frugal based on the current climate. So yeah, it’s managing the best we can with the current resources available … it’s also competing priorities across the organisation*” (Participant 23).

Multiple participants shared that limited funding also affected their ability to deliver training on all aspects of disaster preparedness. A participant said:

I think the biggest barrier or challenge with any of this disaster management, resilience, and capacity building, is that investment … upfront investment into providing the workforce to teach and train our people and keep them accredited and skilled in what is Emergency Management preparedness, response, and recovery (Participant 13).

Another participant explained how planning disaster training exercises is difficult when hospital networks are required to prioritise both funds and people: “*The managers are under enormous pressure to reduce costs; they (are) also under enormous pressure because they often don’t have enough staff to complete the jobs that they already have*” (Participant 9).

#### Staffing issues in disaster preparedness

Participants shared the difficulties of training hospital network staff on disaster preparedness when staff shortages have affected hospitals across the board. A participant explained: “*There are significant challenges in the fact that people are time poor now, people don’t have enough time. We don’t have enough staff; we’re unable to release staff for training*” (Participant 25). Another participant said that disaster management training is deprioritised because of clinicians’ busy schedules: “*to give them that awareness of disaster management … a 2-hour online course … they’ve got their busy 9 to 5 jobs, it’s just going to get pushed and pushed down there*” (Participant 11).

Some participants argued that disaster preparedness training can be difficult for short-staffed hospital networks because clinical training or matters take precedence. A participant explained:

The biggest challenge we have is staffing constraints. around being able to spend some time and really embed emergency management into those clinical areas when they’ve got so much more that they’re responsible for and that they do training under (Participant 5).

High staff turnover also affected participants attempting to train hospital network staff on disaster preparedness. High turnover resulted in short-staffing and COVID-19 made that worse for clinicians as explained by Participant 21:

Within our emergency department, the stress and workload they went on during the first couple of years meant there has been a large staff turnover, so a lot of that experience has sort of moved on and we are having to also grapple with newer staff who may not have actually gone through the experience of dealing with an actual emergency.

#### Ambiguous relationships with state and national health departments

Hospital networks are set up by state or territory governments and so the networks not only work according to these governments’ policy directives, but are also dependent on health departments of these governments in case additional resources are needed.^11 13^ Participants represented numerous hospital networks, most of which were still recovering from two successive disasters, the 2019–2020 Black Summer bushfires and the 2020–2022 COVID-19 pandemic, which they suggested altered their relationships with state and national health departments, creating confused expectations on both sides. Participants argued that the top-down approach replaced the traditional bottom-up approach of disaster response. A participant argued that prior to these events, the principal foundation of disaster response was that it be locally led, which had a shift in the past few years:

Even if you are asking for assistance, you’re asking for support, you’re not asking the state or the national groups to take over. You’re asking for support from them … I feel like COVID’s flipped everything. You know, we were beholden to the national level, and we were beholden to the state level, so I feel like it’s kind of undone all of the work we did on those foundational principles (Participant 20).

Another participant voiced a similar opinion:

A lot more items now have to go past the Ministry of Health … I don’t think it’s possibly the pandemic which has changed … the fires did a little bit, then it was exacerbated further with the pandemic. So, I see a lot of the decision making, not the same as it was pre bush fires … a lot of other people involved to make sure that they’re happy and satisfied with what we’re doing in the local health district (Participant 26).

Some participants reported confusion on the supports they could expect from the state health department, or they mentioned requests for supports that went unanswered. A participant complained about the lack of response on continuation of provided supports, indicating:

In terms of what level of support the Department of Health would provide us … I also find that a little bit ambiguous … like 15 years ago, gave everyone satellite phones … we couldn’t get an answer … whether they were going to upgrade them or wanted us to … each emergency department was provided with CBR kits and the Department of Health have never updated them in over 10 years … it’s unclear, is it now the hospital’s responsibility to update … it’s quite frustrating and I’ve actually asked them a number of times (Participant 1).

State health departments can play a role in enhancing disaster preparedness by focusing on the quality of planning. This view was espoused by a participant in the following words:

I think one of the things that would support us in the health service is if the department actually had a policy direction that forced some maturity … what we want them to do is provide us with a very clear minimum mandatory standard and then some levels of quality to go to, to develop maturity in that space and provide us with an evaluation tool that we can evaluate against (Participant 16).

### Solutions to implement hospital disaster risk mitigation measures

The inductive thematic analysis also captured four solutions that can help hospital network disaster managers overcome the four challenges presented above. The solutions were either directly articulated by research participants or were implied through participants’ identification of the underlying issues that are candidates for addressing these challenges. The following potential solutions were identified: capitalising on hospital network staff’s increased interest after disasters, increasing budgetary allocation through evidence of disaster impacts, increasing staff availability for training by facilitating participation and building accountability in network and government relationships.

#### Capitalising on hospital network staff’s increased interest after disasters

A participant experienced in responses to multiple disasters shared that although on normal occasions there is limited attention paid to disaster preparedness, a window of opportunity opens right after a disaster, when hospital network staff’s interest in disaster preparedness is high and that is when commitment to preparedness measures can be mobilised. The participant explained the window of opportunity in the following words:

The best time to get anything done in ‘Emergency land’, is immediately after a disaster has just finished … so if you can strike while the iron’s hot, virtually straight after a debrief, then it’s great, because people are totally committed. They want to be involved, they want to change things, they want things to work better. As that timeline lengthens away from when the incident occurred, the keenness to fix the issues dwindles … people start to forget just how bad it was. The mind is great thing in working in that space, I suppose, and then people start going; well, we haven’t got the resources to look at that right now (Participant 26).

#### Increasing budgetary allocation for preparedness through evidence of disaster impacts

A participant indicated that although hospital networks operating on limited budgets can deny investments on disaster preparedness measures, it becomes difficult for the networks to deny investments when there is irrefutable evidence of prior disasters’ impacts. The participant shared how investments on preparedness measures can be attracted with the help of prior events’ examples and provided an example of their network’s proposal aimed at addressing the risk of post-disaster communication disruption (announced by code yellow in hospitals):

We need to be able to justify the spending of $200 000 on telecommunications, and for me to justify that I need a list of events … one part of my risk assessment now is I’m compiling 2–3 years’ worth of notifications to ICT (Information, Communication and Technology), 3 years’ worth of code yellows, ICT outage records, to justify the cost (Participant 7).

#### Increasing staff availability for disaster training by facilitating participation

An important part of research participants’ mandate was to train hospital network staff on disaster response; However, Networks’ clinical staff reported that they were stretched thin, compromising their ability to attend disaster trainings. Participants either recommended in-person training to encourage clinicians’ attendance or identified challenges that limit attendance. A participant shared that their hospital network conducts disaster preparedness training in person to ensure that trainees are not distracted by clinical matters and they acquire the skills that can help them in catastrophic events:

I try and make sure that I still go out to the facilities and do my exercises face-to-face … once you’re in the room, I’ve got them. If they’re on Skype, they’re on MS Teams. They can be like ‘Oh sorry have to duck out … just got an emergency’, they say ‘I’ll be back in a minute’, they disappear, and you don’t see them until next training session which may be up to a year’s duration (Participant 26).

Participants also identified two issues that limit clinicians’ attendance in disaster training exercises; the excessive load of clinical training and the high cost of back-filling clinicians participating in disaster training. Participants implied that by addressing these challenges, hospital networks can get a higher percentage of their clinicians prepared for disasters and their impacts on patients. Although clinical training is essential to the core purpose of hospital networks, a balance between clinical and disaster training can be negotiated to build clinicians’ capacities in both functions. A participant highlighted the need to assess the load of clinical training to find that balance:

We would love to run more exercises or more trainings within some of those areas, (but) to … get clinical staff off the floor … then invest in that training, when there is so much more that they already have to do within their own clinical training (Participant 8).

Disaster simulations are demonstrably effective in building clinicians’ capacities in disaster response.^31^,^37^ However, clinicians can only participate if their roles are back-filled to enable continuity of care for hospital network patients. Networks often conduct simulation exercises using a tool (the Emergo Train System),^31 32 35 36 38^ which facilitates clinicians’ capacity building on mass casualty management using plastic cards (known as ‘gubers’) as simulated patients. A participant emphasised why their network would not be able to simulate a disaster to train their clinicians, without the essential investment in arranging temporary staff to fill participating clinicians’ roles:

Emergo, it’s just so expensive because you’ve got to use clinicians to make those decisions about patients, those little gubers, and then you’ve got to backfill those clinicians (Participant 8).

#### Building accountability in hospital network and state and national government relationships

Hospital networks are dependent on state and federal government support when disaster response requires resources beyond their capacities, and different levels of governments are dependent on hospital networks for targeted risk reduction or response because of their knowledge of local risks and response needs.^12^ This interdependency can be better managed by stipulating the supports that networks and governments should both provide and be accountable for. A participant said that the network-government coordination can be improved by achieving clarity on the precise support that state or federal governments’ health departments can provide to hospital networks and vice versa:

There is also a challenge with understanding the ambiguity of accountability between the Department of Health and hospital, and it seems to be a floating line, and I think something that would be good, would be some more precision in that (Participant 1).

## Discussion

### Summary of key results

Participants shared the difficulties of convincing hospital network staff on the importance of preparedness initiatives. Their elaborated reasons included staff being time poor, with, at times, less-than-optimal interest in preparedness and limited availability of funds. Participants discussed the lack of clear expectations between their hospital networks and state and national health departments, which impacted on their ability to understand the support structures that health departments can provide. The emergent themes overlapped as constrained budgets not only restricted training opportunities but also limited staff availability. Staff shortage, in turn, reduced their availability for disaster training; and when there are not enough funds or staff to spare for training, the executive or staff may lose interest in disaster preparedness.

The participants provided solutions. The identified solutions included (1) Capturing hospital network staff’s interest in preparedness after a disaster when they want to be ready for the next disaster, (2) Attracting funding for preparedness through evidence of prior disasters’ impacts, (3) Facilitating clinicians’ participation in disaster training by reducing their clinical training load, backfilling their role or by offering in-person sessions and (4) Determining the exact nature of accountable working relationships between hospital networks and state/federal governments.

### Interpretation

Australia is a disaster-prone country where hospitals in locations, such as Ballina, Numurkah, Cairns, Tara, Batlow and Tumut have been directly affected by climate change-driven disasters.^4 5 7 8 39^ This trend is highly likely to accelerate as an increase in climate change-driven disasters is predicted.^9^ Although Australian hospital networks have shown remarkable logistical preparedness, gaps remain in preparedness for mass casualty incidents and emergency evacuations.^14^ Patients dependent on Australian hospitals may face increased post-disaster mortality and morbidity risks if such gaps are not closed. An important challenge to preparedness identified by this research was the shortage of staff, which limits their availability for training sessions. Lack of training can compromise the continuation of care for hospital patients, increasing their mortality and morbidity risks. According to the Australian Institute of Health and Welfare, a national agency collecting health statistics, there are over 400 000 full-time equivalent healthcare workers in public hospitals and 82% of healthcare occupations had shortages in 2023.^40^ A study found high levels of nurse burnout after COVID-19; nurses alone form 42% of the cumulative hospital workforce.^40 41^ This current study recommends back-filling clinicians’ roles and reducing their clinical training load to allow them to attend in-person disaster trainings. It also recommends evidence-based budget allocation requests for disaster preparedness measures, as hospital network budgets can also be limited. In 2021–2022, Australian public hospitals were run at the cost of AUD$77.2 billion; 55% of that was borne by state and territory governments,^42^ which are running deficits in their budgets post the COVID-19 lockdown period. These statistics provide a context to research participants’ expressed hardships in arranging staff for disaster training and investments for disaster preparedness measures. Reduction in clinical training load might not be possible, or additional staff might not always be available to backfill clinicians nominated for disaster training, and so innovations such as disaster preparedness champions’ routine guidance to clinicians can also be thought of as alternative solutions.

This study’s identification of the challenge of budgetary constraint aligned with the finding of a qualitative study of Australian hospital staff’s experiences in both disaster planning and responses to extreme weather events, which also revealed lack of funds as a challenge limiting disaster training.^43^ Although Australia has a relatively well-funded and staffed health system, with robust and operationally sound infrastructure,^1^ high demand for healthcare creates competition for all resources, which may leave less room for the under-prioritised function of disaster preparedness. According to the Organisation for Economic Co-operation and Development (OECD) indicators, the ratio of emergency department presentations per hundred people was higher in Australia in 2021 compared with a range of OECD countries including Italy, Belgium, New Zealand, Finland, Switzerland, Germany, Sweden, the Netherlands and Ireland.^44^ Higher demand for urgent care might require a higher proportion of limited resources, so it may be understandable for participants of this research to report challenges of limited funds and staff availability.

The projected increase in climate change-driven disasters requires a proportional increase in investments on preparedness measures. Disasters have had devastating financial consequences for Australian hospital networks. However, disaster losses are known to be substantially reduced through timely investments. The second National Action Plan of the Australian Government’s National Emergency Management Agency quotes the Insurance Council of Australia’s reporting of a return of AUD$9.6 on every dollar invested in disaster risk reduction.^45^ A more conservative return was estimated by the government of the Australian state of Queensland, which invested AUD$174 million in building resilience of 423 public assets in disaster-prone regions. Those assets were re-impacted by 44 different disasters, yet the financial burden was negligible, saving the tax payers an estimated AUD$397.5 million.^46^ For every AUD$1 that the Australian state of Queensland spent on risk reduction, they avoided spending AUD$2.28 on recovery. A higher ratio was reported by Porter *et al*,^47^ who calculated that each dollar of disaster mitigation grants disbursed by the US Government saved six dollars after disasters. Positive rates of return reported by these publications are generally related to investments in preparedness of specific areas or populations, for example, the disaster-prone area in Queensland where 423 public assets are located. Although these investments were not tied to specific events, subsequent disasters and their reduced recovery costs proved the efficacy of these investments. Although Australian hospital networks’ budgetary constraints are understandable, the benefits of investing in preparedness measures would likely outweigh the costs incurred, and the benefit of saving lives would be on top of that economic gain. A survey-based study of challenges to American community hospitals’ disaster preparations suggested that high COVID-19 mortality in the US might be attributable to financial challenges to disaster preparedness, as a decline in funding had preceded the pandemic.^48^ This example underscores the need for timely investment in disaster prevention, to yield tangible returns for disaster-ready hospital networks.

Participants of this study, consistent with other international research,^48^,^50^ saw limited interest in disaster preparedness as a significant impediment. The findings reported by this Australia-wide study aligned with the findings of a study of ten Mexican hospitals previously affected by extreme weather events. The Mexican research studied the challenges that were faced when the hospitals there tried to implement WHO’s Hospital Safety Index, a checklist of preparedness measures,^51^ and reported ‘lack of commitment from hospital staff’, ‘lack of experts’, ‘lack of space for surge’ and ‘lack of funds’.^50^ These Mexican hospitals had already been affected by hurricanes and heavy rains, yet the hospital staff’s lack of interest and commitment persisted, except in the immediate aftermath of a disaster.^50^ This finding resonated with a solution to overcome the lack of interest, captured by this research. The participant who articulated this solution emphasised how interest in disaster preparedness dwindles as the disaster memory fades in hospital network staff’s minds, which in turn precludes allocation of resources to preparedness. This research highlights the importance of understanding the timing of network staff’s increased interest in preparedness and capitalising on that window of opportunity to get network resources committed for hospital disaster preparedness. Affected hospital network staff and patients’ video recorded impact statements can possibly rekindle network staff’s interest in preparedness, even after it has dwindled. Although the solution of capitalising on the brief window can overcome the existing challenge, an increase in general interest should be advocated because disasters wreak the most havoc when hospitals are underprepared. Disasters, such as the COVID-19 pandemic, exact the highest toll on hospital patients because lack of hospitals’ foresight can constrict preparedness. If a reactive approach to funding preparedness only immediately after disasters is adopted, then hospital patients would remain vulnerable to disasters that strike after a long time.

This study’s findings aligned with a systematic review of disaster risk management challenges of hospitals, which identified challenges such as inadequate staff and financial resources, lack of commitment or motivation, and the absence of a culture of preparedness and political will, particularly among the hospital leadership ranks.^49^ Challenges to hospital disaster preparedness are interrelated and mutually reinforcing, which creates the need to break the cycle, by identifying circuit breakers that trigger a virtuous cycle by directly addressing a particular challenge and indirectly facilitating the overcoming of various other challenges. For example, increasing hospital network leadership’s interest in preparedness can later facilitate both staff availability and budget allocations for disaster preparedness. Under-preparedness increases morbidity and mortality risks for hospital patients as exemplified by shortages of personal protective equipment, ventilators and negative pressure isolation rooms during the COVID-19 pandemic.^52^ A holistic view of interrelated challenges should be taken to consider unprecedented innovations that can trigger a positive cascading effect to address all challenges. The proposed solution of capitalising on the short window of increased interest after a disaster can also help hospital disaster managers obtain staff and funding allocations and establish clearly understood reciprocal relationships with external stakeholders. A day before the landfall of Hurricane Irene, New York University’s Langone Medical Centre was ordered to pre-emptively evacuate all of its patients in the Neonatal Intensive Care Unit (NICU), but the hospital lacked a directory of regional NICUs that they could quickly call and arrange transfers with. There was increased interest in preparing such a directory immediately after Hurricane Irene and so 14 months later, when Hurricane Sandy forced Langone Medical Centre to evacuate, the directory was immediately employed to initiate transfers of all infants in the NICU.^53^ This evidence of proposed solutions’ implications for practice highlights the need for future research on preparedness initiatives that hospitals undertake immediately after disasters.

A limitation of this research was the potential for social-desirability bias, whereby participants could have reported substantial preparedness within their hospital networks instead of initiating frank discussions about gaps, challenges and impediments. This bias was mitigated by acknowledging their achievements and reframing the questions to keep the interviews focused on the challenges that they faced when reducing disaster risks for hospital networks.

## Conclusion

Prior research revealed that Australian hospital networks have reduced the risks of post-disaster logistical issues by investing in substantial backups of various resources, but certain challenges restricted implementation of other important preparedness measures. This research sought to identify the exact challenges and explore their solutions. Although it is not possible to reduce all disaster risks, efforts must be made to reduce most risks of adverse impacts of disasters. This research identified the challenge of hospital network staff’s limited interest in preparedness but also reported how a participant worked around that by obtaining staff’s commitment to preparedness measures immediately after a disaster, when staff’s interest in the matter had not yet dissipated. Similarly, the study found budgetary constraints and staffing issues as challenges to disaster preparedness, but it also reported evidence-based budget allocation requests and back-filling for in-person training or balancing clinical and disaster training as potential solutions.

Finally, the participants reported that there were unclear expectations between hospital networks and state and federal governments, and this study captured a participant’s recommendation of clear articulation of an accountable relationship between these entities. We uncovered challenges to hospital networks’ disaster preparedness and presented solutions to support networks in overcoming those challenges. Although the reported solutions present ways to work around the identified challenges, substantial efforts are needed to systematically remove all challenges, as under-preparedness can have devastating consequences for patients of hospitals affected by disasters.

## Supplementary material

10.1136/bmjopen-2025-106500 online supplemental file 1

## Data Availability

No data are available.
