# Supplementary material for: Disaster preparedness of Australian hospital networks: a qualitative study with key actors
Source: BMJ Open. 2026 Feb 22;16(2):e106500. doi: 10.1136/bmjopen-2025-106500 (PMC12927313; doi:10.1136/bmjopen-2025-106500)
Supplement: Supplementary file 2 [file bmjopen-2025-106500.reviewer_comments.pdf]

PEER REVIEW HISTORY

BMJ Open publishes all reviews undertaken for accepted manuscripts. Reviewers are asked to complete a checklist review form and are provided with free text boxes to elaborate on their assessment. These free text comments are reproduced below.

ARTICLE DETAILS

Title (Provisional)

Disaster preparedness of Australian hospital networks: a qualitative study with key actors

Authors

Naru, Faran Shoaib; Churruca, Kate; Long, Janet C; Sarkies, Mitchell N; Braithwaite, Jeffrey

---

VERSION 1 - REVIEW

**Reviewer**	**1**
**Name**	**Augusterfer, Eugene**
**Affiliation**	**Mass General Brigham Inc, Psychiatry**
**Date**	**09-Oct-2025**
**COI**	**None**

Thank you for this excellent paper on this important, but often overlooked, area! Well done.

**Reviewer**	**2**
**Name**	**Lamine, Hamdi**
**Affiliation**	**Universita degli Studi del Piemonte Orientale Amedeo Avogadro Scuola di Medicina, CRIMEDIM**
**Date**	**12-Oct-2025**
**COI**	**None**

I am extremely sorry for the delay in reviewing your manuscriot, I have gone through some personal events that delayed all my schedules.

I have with interest your manuscript and it reads very well, I have 2 minor comments

In line 222 it is not clear the contribution of the co-authors in the thematic analysis.

At the first part of the results section the presentation looks very quantitative (with percentages and representation concerns).

Other than that I like your manuscript very well.

Reviewer **3**

Name **Abrahams, Jonathan**

Affiliation **Monash University, Monash University Disaster Resilience Initiative, Monash University Accident Research Centre**

Date **15-Dec-2025**

COI **None**

Thank you to the authors for presenting the challenges and solutions to address the challenges for hospital networks in Australia. Herebelow are proposed areas for improvement.

Authors are using related terms interchangeably and often without describing them or demonstrating how these terms have different meanings and applications. There are considerable differences between disaster risk reduction, disaster management, mitigation, preparedness etc. In particular, disaster risk reduction (as described in the Sendai Framework), is a very broad concept - of which preparedness is one part. As your paper is mostly focused on preparedness, it is proposed to use one term - 'preparedness' - and define it in relation to the other terms.

It is also proposed to change the title of the paper accordingly. Similarly, the use of the term "stakeholders" may not be appropriate because the participants are (not just stakeholders but) key actors in hospital preparedness.

It would be helpful if the authors could share more details about the participants (in addition to their designated roles and state-based locations). For example, their experience of disasters or whether the participants are in high risk areas for disasters.

Similarly, it would be helpful to for the authors to indicate the frames of reference for hazards or disaster that participants are referring to? For example, are their responses based on natural hazards or COVID-19 or everything?

The use of terms such as 'unprecedented' and 'unpredictable" disasters should be reviewed. Many experts forewarned of pandemics and disasters may have precedents. Effective risk assessment can identify these risks and enable effective risk management.

It would be helpful for the authors to explain whether the references to research on returns on investment refer to analysis of particular events (where analysis is focused on an actual event, i.e. the risk has materialised) or to the whole area/population at risk (where there is a need to determine when and where to invest) which extends the denominator considerably.

It would be most useful for the authors to expand on the section on the presentation of solutions by interviewees. This is a golden opportunity to present achievements and success

stories from the participants, and provide practical evidence to support the proposed solutions.

Please check in-text citations and references to WHO and PAHO publications. They are incorrect, probably due to the software being used.

**Reviewer**	**4**
**Name**	**Harris, Myles**
**Affiliation**	**UCL, Institute for Risk and Disaster Reduction**
**Date**	**19-Dec-2025**
**COI**	**None**

Thank you for the opportunity to review this article, which I enjoyed reading.

Question 9 in the COREQ checklist refers to content analysis, whereas the article refers to thematic analysis. Thematic analysis appears to be more appropriate, so presumably this is a typo on the checklist.

The abstract provides a succinct and accurate overview of the article.

Lines 64-65 – this claim requires substantiating evidence and rationale, especially given the research participants represented of five out of eight states/territories in Australia. This should be included in the limitations section of the article.

There appears to be some inconsistency with, "…low frequency of disasters…" [line 55] and, "Australia is also a disaster-prone country…" [lines 87-88], which is referred to elsewhere in the article.

Line 150 – Consider if the phrase 'natural disaster' should be amended to something like, "disasters caused by vulnerability to natural hazards." See 'Disasters by choice\ (Kelman, 2020) and UNDRR position on natural disasters (https://www.undrr.org/our-impact/campaigns/no-natural-disasters).

Use of a pilot study demonstrates robustness of research design.

Use of Figure 1 and Table 1 provide clarity to the participants included in this research project.

The findings are presented clearly in alignment with good practice, and there is a logical structure to the themes. Furthermore, the thematic structuring links with the thematic data analysis, which demonstrates robustness.

In the discussion, the findings of this research are situated in the evidence-base accurately. The limitation of social-desirability bias was appropriately mitigated.

Consider including greater depth in the discussion/conclusion about the implications for practice this research has, and recommendations for future research to build on this foundation.

The COREQ checklist had sufficient detail to provide context to the research process used for this project.

Overall this is a significant research project that, with some minor amendments, will be a valid publication.

VERSION 1 - AUTHOR RESPONSE

	Manuscript ID bmjopen-2025-106500	
	**Title: Disaster preparedness of Australian hospital networks: a qualitative study with key actors**	
	**Reviewer 1's comment**	**Authors' Responses**
1	Reviewer: 1<br>Dr. Eugene Augusterfer, Mass General Brigham Inc<br>Comments to the Author:<br>Thank you for this excellent paper on this important, but often overlooked, area! Well done.	Much appreciated.
	**Reviewer 2's comments**	**Authors' Responses**
1	Reviewer: 2<br>Dr. Hamdi Lamine, Universita degli Studi del Piemonte Orientale Amedeo Avogadro Scuola di Medicina<br>Comments to the Author:<br>I am extremely sorry for the delay in reviewing your manuscriot, I have gone through some personal events that delayed all my schedules.<br>I have with interest your manuscript and it reads very well, I have 2 minor comments<br>In line 222 it is not clear the contribution of the co-authors in the thematic analysis.	Co-authors' contribution to the thematic analysis was clarified in the following words, added to the manuscript:<br>"The thematic analysis was conducted by the first author but KC, JL and JB continuously evaluated and classified the findings as the analysis proceeded. All authors formally ratified the final themes emerging from the analysis."
2	At the first part of the results section the presentation looks very quantitative (with percentages and representation concerns).<br>Other than that I like your manuscript very well.	The quantitative information is intended to complement the qualitative results, to inform the reader on participants' geographic locations and roles in hospital management.
	**Reviewer 3's comments**	**Authors' Responses**

1	Reviewer: 3<br>Dr. Jonathan Abrahams, Monash University<br>Comments to the Author:<br>Thank you to the authors for presenting the challenges and solutions to address the challenges for hospital networks in Australia. Herebelow are proposed areas for improvement.<br><br>Authors are using related terms interchangeably and often without describing them or demonstrating how these terms have different meanings and applications. There are considerable differences between disaster risk reduction, disaster management, mitigation, preparedness etc. In particular, disaster risk reduction (as described in the Sendai Framework), is a very broad concept - of which preparedness is one part. As your paper is mostly focused on preparedness, it is proposed to use one term - 'preparedness' - and define it in relation to the other terms.	The term 'disaster preparedness' has now replaced 'disaster risk reduction', 'disaster management', and 'mitigation' throughout the manuscript and its definition was added in the following words:<br><br>"United Nations Office for Disaster Risk Reduction (UNDRR) defines disaster preparedness as "*the knowledge and capacities developed to effectively anticipate, respond to and recover from the impacts of likely, imminent or current disasters*""
2	It is also proposed to change the title of the paper accordingly. Similarly, the use of the term "stakeholders" may not be appropriate because the participants are (not just stakeholders but) key actors in hospital preparedness.	That is a good proposition. The title was updated as follows: "Disaster preparedness of Australian hospital networks: a qualitative study with key actors"
3	It would be helpful if the authors could share more details about the participants (in addition to their designated roles and state-based locations). For example, their experience of disasters or whether the participants are in high risk areas for disasters.	The following text was added to explain how participants are in high-risk areas for disasters:<br><br>"Participants of different states prepare their hospital networks for different hazards, for e.g. hospitals in northern Queensland are located in high cyclone risk areas, those in northern New South Wales are in high flood risk areas. The risk of bushfire damage however is faced by hospitals across Australia."<br><br>Mentioning individual participants' experiences of specific disasters would easily identify them, compromising both their

		privacy and the confidential nature of the research. The ethics approval of this research as well as the Participant Information and Consent Forms returned by all participants requires complete de-identification, so an overview of all participants' experiences of different disaster types is provided in response to the next comment.
4	Similarly, it would be helpful to for the authors to indicate the frames of reference for hazards or disaster that participants are referring to? For example, are their responses based on natural hazards or COVID-19 or everything?	The following text was added to explain that participants were referring to multiple hazards in their responses:<br><br>"Most hospital networks participating in this research had been affected by bushfires in 2019-20 and then COVID-19 in succession. A number of participating hospital networks had also been affected by 2022 or earlier floods, therefore almost all participants' responses were informed by their practical experiences in disaster response."
5	The use of terms such as 'unprecedented' and 'unpredictable" disasters should be reviewed. Many experts forewarned of pandemics and disasters may have precedents. Effective risk assessment can identify these risks and enable effective risk management.	The terms ''unprecedented' and 'unpredictable' disasters were removed from the manuscript.
6	It would be helpful for the authors to explain whether the references to research on returns on investment refer to analysis of particular events (where analysis is focused on an actual event, i.e. the risk has materialised) or to the whole area/population at risk (where there is a need to determine when and where to invest) which extends the denominator considerably.	The references to research on returns on investments referred to whole area/population. The following text was added in the manuscript to clarify that:<br><br>"Positive rates of return reported by these publications are generally related to investments in

		preparedness of specific areas or populations, for e.g. the disaster prone area in Queensland where 423 public assets are located. Although these investments were not tied to specific events, subsequent disasters and their reduced recovery costs proved the efficacy of these investments.”
7	It would be most useful for the authors to expand on the section on the presentation of solutions by interviewees. This is a golden opportunity to present achievements and success stories from the participants, and provide practical evidence to support the proposed solutions.	Practical evidence of the proposed solutions was added in the following words to expand on the presentation of solutions proposed by interviewees:<br><br>“The proposed solution of capitalizing on the short window of increased interest after a disaster, can also help hospital disaster managers obtain staff and funding allocations, and establish clearly understood reciprocal relationships with external stakeholders. A day before the landfall of Hurricane Irene, New York University’s Langone Medical Center was ordered to pre-emptively evacuate all of its patients in the Neonatal Intensive Care Unit (NICU) but the hospital lacked a directory of regional NICUs that they could quickly call and arrange transfers with. There was increased interest in preparing such a directory immediately after Hurricane Irene and so 14 months later when Hurricane Sandy forced Langone Medical Center to evacuate, the directory was immediately

		employed to initiate transfers of all infants in the NICU (54). This evidence of proposed solutions' implications for practice highlights the need for future research on preparedness initiatives that hospitals undertake immediately after disasters."
8	Please check in-text citations and references to WHO and PAHO publications. They are incorrect, probably due to the software being used.	The citation was corrected, so that it displays the organisation's acronym properly.
	**Reviewer 4's comments**	**Authors' Responses**
1	Reviewer: 4<br>Mr. Myles Harris, UCL<br>Comments to the Author:<br>Thank you for the opportunity to review this article, which I enjoyed reading.<br><br>Question 9 in the COREQ checklist refers to content analysis, whereas the article refers to thematic analysis. Thematic analysis appears to be more appropriate, so presumably this is a typo on the checklist.	The response to question 9 in the COREQ checklist was corrected by providing the page number in the manuscript on which our 'thematic' analysis is explained.
2	The abstract provides a succinct and accurate overview of the article.<br><br>Lines 64-65 – this claim requires substantiating evidence and rationale, especially given the research participants represented of five out of eight states/territories in Australia. This should be included in the limitations section of the article.	What the reviewer saw as a limitation is actually a strength of the research. The following text was added to the strengths and limitations section to provide the substantiating evidence:<br><br>"Participants represented six out of eight Australian states and territories, and these six states and territory contain 98.4% of Australia's population."
3	There appears to be some inconsistency with, "…low frequency of disasters…" [line 55] and, "Australia is also a disaster-prone country…" [lines 87-88], which is referred to elsewhere in the article.	Line 55 (the start of conclusion subsection of the abstract) was edited as follows, to address the inconsistency and underscore the disaster-prone nature of Australia:<br><br>"Disasters, although infrequent are known to occur and can be

		catastrophic, yet those working in hospital network disaster risk reduction roles; encounter limited availability of wider staff for training and low interest in disaster planning.”
4	Line 150 – Consider if the phrase ‘natural disaster’ should be amended to something like, “disasters caused by vulnerability to natural hazards.” See ‘Disasters by choice\ (Kelman, 2020) and UNDRR position on natural disasters (https://www.undrr.org/our-impact/campaigns/no-natural-disasters).	Reviewer’s words were used to make that correction. We are grateful to the reviewer for articulating the precise explanation of such disasters.
5	Use of a pilot study demonstrates robustness of research design.<br><br>Use of Figure 1 and Table 1 provide clarity to the participants included in this research project.<br><br>The findings are presented clearly in alignment with good practice, and there is a logical structure to the themes. Furthermore, the thematic structuring links with the thematic data analysis, which demonstrates robustness.<br><br>In the discussion, the findings of this research are situated in the evidence-base accurately. The limitation of social-desirability bias was appropriately mitigated.<br><br>Consider including greater depth in the discussion/conclusion about the implications for practice this research has, and recommendations for future research to build on this foundation.<br><br>The COREQ checklist had sufficient detail to provide context to the research process used for this project.<br><br>Overall this is a significant research project that, with some minor amendments, will be a valid publication.	The text added in response to Reviewer 3’s comment 7 added depth to the discussion section by providing an example that highlighted this research’s implication for practice and a recommendation for future research was also provided.

VERSION 2 - REVIEW

Reviewer 3

Name Abrahams, Jonathan

Affiliation Monash University, Monash University Disaster Resilience Initiative, Monash University Accident Research Centre

Date 25-Jan-2026

COI

The changes made to the paper in response to the reviewers are appropriate, appreciated and strengthen the paper. It would be ok to take the view that hospital emergency managers have responsibilities for taking ex-ante action that is more preventative and precede preparedness (although this can be a semantic and largely inconsequential argument). Given the focus of the manuscript on preparedness, this has been well accounted for in the amendments. Thank you. Best wishes.
